# Association between ABO blood groups and risk of coronavirus disease 2019: A protocol for systematic review and meta-analysis

**DOI:** 10.1097/MD.0000000000021709

**Published:** 2020-08-14

**Authors:** Nanyang Liu, Tingting Zhang, Lina Ma, Huican Wang, Hao Li

**Affiliations:** a Xiyuan Hospital, China Academy of Chinese Medical Sciences, Beijing; b College of First Clinical Medicine, Shandong University of Traditional Chinese Medicine, Jinan, Shandong Province, P.R. China.

**Keywords:** ABO blood groups, coronavirus disease 2019, severe acute respiratory syndrome Coronavirus 2, protocol, systematic review and meta-analysis

## Abstract

**Background:**

The 2019 Coronavirus Disease (COVID-19) pandemic has threatened millions of people worldwide. Growing evidence suggests that the ABO blood type contributed to the susceptibility of COVID-19, but the results are controversial. The major objective of this systematic review and meta-analysis study is to investigate the impact of ABO blood group on COVID-19 pneumonia.

**Methods:**

Two independent reviewers searches the databases of the China Biology Medicine disc, China National Knowledge Infrastructure, China Science and Technology Periodical Database, Wanfang Database, PubMed, Embase, and Web of Science from the date of conception to June 30, 2020. We will manually search for gray literature, such as meeting records and dissertations. Two independent reviewers will screen studies that meet the criteria, extract data, statistical data, and assess the risk of bias. The dichotomous variable will calculate the odds ratio and the corresponding 95% confidence interval. Heterogeneity between included studies will be assessed by heterogeneity *χ*^2^ tests and *I*^2^ index. The forest plots will be used to describe the pooled results. The Begg rank correlation test or Egger linear regression test will be performed to quantize the publication bias.

**Discussion:**

This study will provide high-quality evidence to evaluate the contribution of the ABO blood group in COVID-19 pneumonia infection.

**PROSPERO registration number:**

CRD42020195615

## Abstract 2

Supplemental Digital Content is available in the text

## 1 Introduction

Since the outbreak of the 2019 Coronavirus Disease (COVID-19) in Wuhan, China, at the end of December 2019, the virus has spread rapidly throughout the country.^[1]^ To date, more than 200 countries/regions in the world reported COVID-19 cases which have seriously threatened human life.^[2]^ Due to its widespread epidemic and high fatality rate, the WHO declared it an international public health emergency. Studies have reported that the current COVID-19 pandemic caused the severe acute respiratory syndrome coronavirus 2 (SARS-CoV-2) is genetically similar to the SARS-CoV that caused the severe acute respiratory syndrome (SARS) outbreak in 2003, and both through ACE2 as a carrier to enter the cell. Recently, the next-generation sequencing of SARS-CoV-2 showed a 99.98% sequence identity in 9 patients. Epidemiology reports on risk factors for SARS-CoV-2 susceptibility, including age, gender, and chronic disease.^[3,4]^ Researchers from all over the world are actively engaged in searching biomarkers that can predict this disease.

Several studies are extremely interested in the ABO blood type. It is established knowledge nowadays that the importance of ABO blood type in blood transfusion and clinical transplantation. Multiple studies demonstrate that the ABO blood group is a pivotal independent risk factor for cardiovascular disease and venous thromboembolism.^[5]^ In particular, the risk of thrombosis in blood group O is significantly reduced compared to non-O blood groups. Recent data have defined the biological mechanism by which ABO regulates the risk of thrombosis.^[6–8]^ Given the increasing evidence that COVID-19 is associated with severe coagulopathy^[9]^ and microthrombi distributed through the pulmonary vascular system from acute respiratory distress syndrome,^[10]^ the ABO blood group is associated with the susceptibility of COVID-19 is of particular concern.

A recent study in China compared COVID-19 patients with the general population found an association between the ABO blood group and SARS-CoV-2 infection status.^[11]^ However, another study is not supported by this evidence.^[12]^ This study will systematically review the current evidence, aiming to provide clarity surrounding the role of the ABO blood type in patients with COVID-19.

## 2 Methods

The systematic review and meta-analysis have been registered in PROSPERO (registration number: CRD42020195615). We carry out the protocol according to the Preferred Reporting Item for Systematic Review and Meta-analysis Protocol (PRISMA-P) statement^[13]^ Supplemental Digital Content (Additional file 1). If there get any amendments to the protocol, the registration information will be updated.

### 2.1 Inclusion criteria

#### 2.1.1 Type of participant

(1) participants who were documented to have SARS-CoV-2 infection, and for whom an ABO typing.
(2) health control participants were recorded with ABO blood type. There are not any restrictions on gender, age, race, or any comorbidities.

#### 2.1.2 Type of exposure

ABO blood type distribution in all participants that regardless of testing time and methods.

#### 2.1.3 Type of outcome measurements

The main outcome is to investigate the relationship between ABO blood type and COVID-19 susceptibility. The secondary outcome is to evaluate the prognosis of COVID patients under the ABO blood group.

#### 2.1.4 Type of studies

Observational studies will be included. The sample size, blood type detection method, follow-up time, publication language or publication status will not be limited.

##### 2.1.4.1 Exclusion criteria

Exclusion criteria are

(1) results that cannot be pooled through calculation.
(2) case reports, case series, duplicate reports.
(3) the full text of the study could not be available.

### 2.2 Databases and search strategy

Two independent reviewers search the databases of the China Biology Medicine disc, China National Knowledge Infrastructure, China Science and Technology Periodical Database, Wanfang Database, PubMed, Embase, and Cochrane Library from the date of conception to June 30, 2020. Search terms are: “blood type”, “blood groups”, “ABO”, “novel coronavirus infected pneumonia”, “COVID-19”, “Corona Virus Disease 2019”, “NCP”, “2019-nCOV”. The search words in the Chinese databases are translations of the above words. We will search for the latest research references to obtain potentially relevant articles.

### 2.3 Data collection and analysis

#### 2.3.1 Study selection

**Figure 1:**
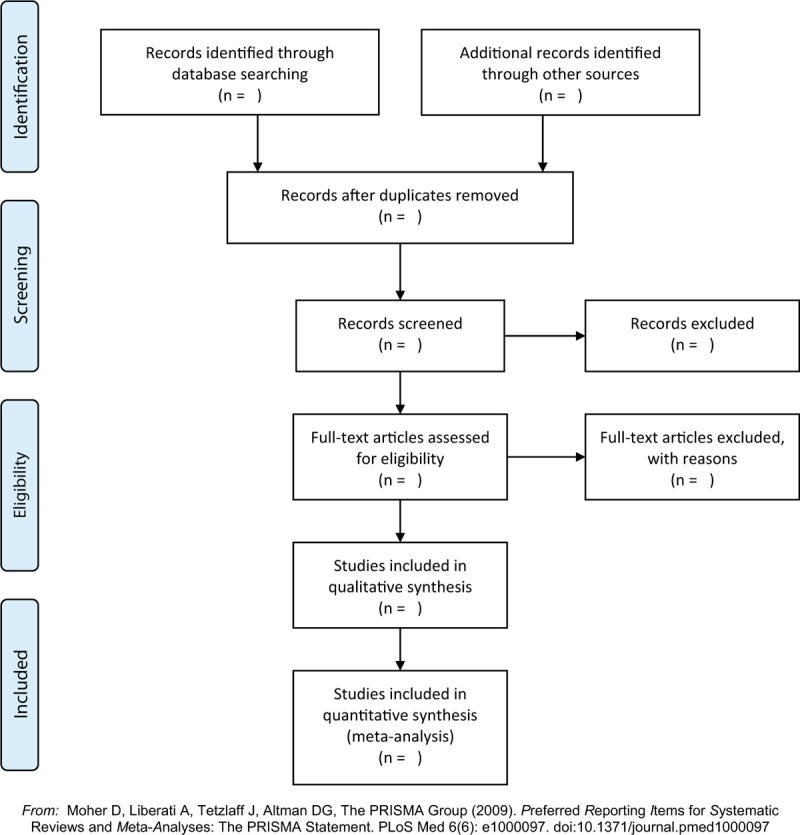
The PRISMA flow chart.

We will export the records retrieved from the database to EndNote X9 software to detect duplicate research. After removing the duplicates, the 2 reviewers will independently check them by reading the title and abstract according to the eligibility criteria. If a study may qualify, the full text will be generated and independently reviewed by 2 reviewers. As for the unverifiable literature, it will be supported by the discussion of the 2 reviewers. If they cannot achieve an agreement, a third reviewer will help. If the full text or user data is not available, the author will be contacted. The PRISMA flow chart is shown in Figure 1.

#### 2.3.2 Data extraction

To ensure data integrity and consistency, 2 independent reviewers will use pre-designed tables to extract data from eligible studies. The table includes the following items:

(1) General information: first author, corresponding author, contact information, journal, year of publication, country/region, funding source, research design;
(2) characteristics of participants: age, gender, race, education level, disease stage, and severity;
(3) Research characteristics: sample size, random sequence generation, allocation concealment, blindness, follow-up time;
(4) exposure: ABO blood group classification
(5) outcomes: The main outcome is to investigate the relationship between ABO blood type and COVID-19 susceptibility. The secondary outcome is to evaluate the prognosis of COVID patients under the ABO blood group. This information will be cross-checked by 2 reviewers. Any differences will be discussed and resolved with the third reviewer.

#### 2.3.3 Assessment of the risk of bias

The Newcastle-Ottawa Scale recommended by the Cochrane Collaboration was used to evaluate the methodological quality of the included studies.^[14]^ The evaluation content includes 3 parts: selection, comparability, and exposure/outcome. There are 8 items in this scale, with a total score of 9. Quality assessments were conducted by 2 researchers, any discrepancies existed 2 authors were solved by discussion or consensus.

#### 2.3.4 Statistical analysis

Review Manager software (version 5.3.5) will be used for statistical analysis of the results. The dichotomous variable will calculate the odds ratio and the corresponding 95% confidence interval. Heterogeneity between included studies will be assessed by heterogeneity *χ*2 tests and *I*^2^ index. A rough explanation guide is as follows: 0% to 40% represents mild heterogeneity; 30% to 60% represents moderate heterogeneity; 50% to 90% represents significant heterogeneity, while 75% to 100% Represents significant heterogeneity. When heterogeneity cannot be explained, 1 method of analysis is to pool it into a random-effects model to pooled the results. Otherwise, a fixed-effect model will be used. If quantitative synthesis is not appropriate, we will describe the included studies. The forest plots will be used to describe the pooled results.

#### 2.3.5 Subgroup analysis

We pre-specified the following variables for subgroup analysis: country/region, ethnicity, sample size, and study type. Furthermore, we will consider other subgroup analyses in the study.

#### 2.3.6 Sensitivity analysis

If there is the heterogeneity, we will conduct a sensitivity analysis to test the robustness of the pooled results.

#### 2.3.7 Publication bias

If more than 10 studies met the eligibility criteria, the Begg rank correlation test or Egge linear regression test will be performed to quantize the publication bias.

#### 2.3.8 Quality of evidence

The Grading of Recommendations Assessment, Development, and Evaluation system will be used to estimate the quality of evidence for each result.^[15]^ Each result will be evaluated according to the following 5 aspects: limitations, inconsistency, indirectness, inaccuracy, and publication bias. The grade of each evidence will be defined as high, moderate, low, or very low.

## 3 Discussion

Recent studies suggest that age, gender, and blood type distribution is related to COVID-19 disease susceptibility. Among them, elderly and male patients with COVID-19 are most likely to progress to severe disease.^[1,16]^ Besides, it was also noted that people with blood type A had high susceptibility, while people with blood type O had low susceptibility.^[17]^ It remains unclear whether these causal relationships are established or whether the association is trivial. However, previous studies confirmed that the association between blood type and other coronavirus infections cannot be ignored.^[18]^ As accumulating evidence data floods in every day, blood types should be registered for each infected individual to correlate with larger data sets in the future. Moreover, it should be pointed out that the ABO blood type varies by race,^[12]^ so when comparing infected and uninfected people, this may affect the observed outcome.

To our knowledge, this is the first review to examine the contribution of the ABO blood group to infection with COVID-19 pneumonia. We rate the strengths and limitations of existing evidence through a systematic review and meta-analysis. The protocol will be guided by the PRISMA statement^[19]^ to achieve the highest possible quality in reporting and methodology. The development of this protocol based on the current research design may have limitations, if there get any amendments, the registration information will be updated at PROSPERO.

In conclusion, this systematic review and meta-analysis will serve to explore the susceptibility of the ABO blood group to COVID-19 pneumonia and will help us prepare for future epidemics.

## Author contributions

**Conceptualization:** Nanyang Liu, Hao Li.

**Data curation:** Lina Ma, Tingting Zhang.

**Investigation:** Huican Wang, Nanyang Liu.

**Methodology:** Nanyang Liu, Lina Ma.

**Supervision:** Hao Li.

**Validation:** Lina Ma, Hao Li.

**Visualization:** Nanyang Liu, Lina Ma.

**Writing – original draft:** Nanyang Liu, Tingting Zhang.

**Writing – review & editing:** Hao Li.

## Supplementary Material

Supplemental Digital Content

## References

[1] Wang D Hu B Hu C Clinical characteristics of 138 hospitalized patients with 2019 novel coronavirus-infected pneumonia in wuhan, china. JAMA 2020;323:1061–9. 10.1001/jama.2020.1585 PMC7042881 32031570

[2] Lu R Zhao X Li J Genomic characterisation and epidemiology of 2019 novel coronavirus: implications for virus origins and receptor binding. Lancet 2020;395:565–74. 32007145 10.1016/S0140-6736(20)30251-8 PMC7159086

[3] Fang L Karakiulakis G Roth M Are patients with hypertension and diabetes mellitus at increased risk for COVID-19 infection? Lancet Respir Med 2020;8:e21. 32171062 10.1016/S2213-2600(20)30116-8 PMC7118626

[4] Chen N Zhou M Dong X Epidemiological and clinical characteristics of 99 cases of 2019 novel coronavirus pneumonia in Wuhan, China: a descriptive study. Lancet 2020;395:507–13. 32007143 10.1016/S0140-6736(20)30211-7 PMC7135076

[5] Jenkins PV O’Donnell JS ABO blood group determines plasma von Willebrand factor levels: a biologic function after all? Transfusion 2006;46:1836–44. 17002642 10.1111/j.1537-2995.2006.00975.x

[6] Dunne E Qi QM Shaqfeh ES Blood group alters platelet binding kinetics to von Willebrand factor and consequently platelet function. Blood 2019;133:1371–7. 30642918 10.1182/blood-2018-06-855528

[7] Bowen DJ An influence of ABO blood group on the rate of proteolysis of von Willebrand factor by ADAMTS13. J Thromb Haemost 2003;1:33–40. 12871537 10.1046/j.1538-7836.2003.00007.x

[8] Gill JC Endres-Brooks J Bauer PJ The effect of ABO blood group on the diagnosis of von Willebrand disease. Blood 1987;69:1691–5. 3495304

[9] Fogarty H Townsend L Ni CC More on COVID-19 coagulopathy in Caucasian patients. Br J Haematol 2020;189:1060–1. 32400024 10.1111/bjh.16791 PMC7272907

[10] Mcgonagle DO Donnell J Sharif K Why the immune mechanisms of pulmonary intravascular coagulopathy in COVID-19 pneumonia are distinct from macrophage activation syndrome with disseminated intravascular coagulation. 2020.

[11] Wu Y Feng Z Li P Relationship between ABO blood group distribution and clinical characteristics in patients with COVID-19. Clin Chim Acta 2020;509:220–3. 32562665 10.1016/j.cca.2020.06.026 PMC7832938

[12] Dzik S Eliason K Morris EB COVID-19 and ABO blood groups. Transfusion 2020. 10.1111/trf.15946 PMC7323215 32562280

[13] Shamseer L Moher D Clarke M Preferred reporting items for systematic review and meta-analysis protocols (PRISMA-P) 2015: elaboration and explanation. BMJ 2015;349:g7647. 10.1136/bmj.g7647 25555855

[14] Stang A Critical evaluation of the Newcastle-Ottawa scale for the assessment of the quality of nonrandomized studies in meta-analyses. Eur J Epidemiol 2010;25:603–5. 20652370 10.1007/s10654-010-9491-z

[15] Guyatt GH Oxman AD Vist GE GRADE: an emerging consensus on rating quality of evidence and strength of recommendations. BMJ 2008;336:924–6. 18436948 10.1136/bmj.39489.470347.AD PMC2335261

[16] Arachchillage D Laffan M Abnormal coagulation parameters are associated with poor prognosis in patients with novel coronavirus pneumonia. J Thromb Haemost 2020;18:1233–4. 32291954 10.1111/jth.14820 PMC7262191

[17] Zietz M Tatonetti NP Testing the association between blood type and COVID-19 infection, intubation, and death. medRxiv 2020. 10.1038/s41467-020-19623-x PMC7666188 33188185

[18] Cheng Y Cheng G Chui CH ABO blood group and susceptibility to severe acute respiratory syndrome. JAMA 2005;293:1450–1. 10.1001/jama.293.12.1450-c 15784866

[19] Moher D The LATJ PRISMA Group Preferred reporting items for systematic reviews and meta-analyses: the PRISMA statement. BMJ 2009;339:2535. 10.1136/bmj.b2535 PMC2714657 19622551

